# Exploratory whole-exome analysis of low-density lipoprotein cholesterol and triglyceride response to Mediterranean-style dietary guidance: a focus on plasma lipoprotein pathways

**DOI:** 10.3389/fnut.2026.1827989

**Published:** 2026-07-16

**Authors:** Saba Iordanishvili, Nazibrola Chiradze, Dodo Agladze, Marine Kikvidze, Zaza Khuchua, Vincenzo Lagani, Revaz Solomonia

**Affiliations:** 1 Institute of Chemical Biology, School of Natural Sciences and Medicine, Ilia State University, Tbilisi, Georgia; 2 PhD and Research Department, Petre Shotadze Tbilisi Medical Academy, Tbilisi, Georgia; 3 Diabetes, Endocrine and Metabolism Department; LTD Diacor – The Center for Diabetes, Endocrine and Cardiopulmonary Diseases, Tbilisi, Georgia; 4 Department of Clinical Genetics, Medical Genetics and Laboratory Diagnostic Center, Tbilisi, Georgia; 5 East European University, Tbilisi, Georgia; 6 Division of Biomedical Sciences, King Abdullah University of Science and Technology (KAUST), Thuwal, Saudi Arabia; 7 Ivane Beritashvili Center of Experimental Biomedicine, Tbilisi, Georgia

**Keywords:** gene-diet interactions, LDL-C, lipoprotein metabolism, Mediterranean diet, nutrigenomics, triglycerides, whole-exome sequencing

## Abstract

**Objectives:**

Inter-individual variability in lipid response to dietary modification highlights the critical need for precision nutrition. Current evidence remains fragmented, relying predominantly on restricted candidate-gene studies rather than comprehensive genomic approaches. To bridge this gap, we applied a biologically informed analytical framework to whole-exome sequencing (WES) data to evaluate the nutrigenetics of lipid response in an underrepresented Georgian cohort. Specifically, we explored whether exome-wide and pathway-aggregated genetic variation may contribute to inter-individual differences in LDL cholesterol (ΔLDL-C) and triglyceride (ΔTAG) response to Mediterranean-style dietary guidance.

**Methods:**

A longitudinal study was conducted with 51 dyslipidemic patients who received Mediterranean-style dietary guidance for at least 2 months. High-depth whole-exome sequencing (mean 112×) was performed, and a parallel analytical approach was used: an exploratory exome-wide association analysis to screen for nominal variant-level signals, and a targeted gene-level analysis of the ‘Plasma Lipoprotein Assembly, Remodeling, and Clearance’ pathway to prioritize biologically plausible signals.

**Results:**

The intervention resulted in mean reductions of 33.5 mg/dL in LDL-C and 18.6 mg/dL in TAG. Exome-wide screening identified no variants reaching exome-wide significance. The lowest nominal *p*-values were observed for ΔLDL-C at *KIF6* (*p* = 4.87 × 10^−7^; FDR = 0.122) and for ΔTAG at *SLC35F2* (*p* = 3.08 × 10^−6^; FDR = 0.521). These results were therefore reported only descriptively, without biological interpretation as candidate diet-response loci. In the targeted Reactome pathway analysis, *APOC3* was the top-ranked ΔLDL-C gene-level signal and the only pathway-level result surviving FDR correction (Simes *p* = 7.84 × 10^−4^; FDR = 0.0361), whereas no ΔTAG pathway-level result survived FDR correction. Variant-level inspection showed that the *APOC3* signal was driven primarily by an intronic variant with low model-specific genotype-complete sample size (*N* = 10), supporting interpretation as a hypothesis-generating locus-level signal rather than confirmatory evidence of a gene-diet association.

**Conclusion:**

This exploratory WES study provides a pragmatic framework for pathway-focused nutrigenomic analysis of lipid response in an underrepresented clinical cohort. The results prioritize *APOC3* as a hypothesis-generating LDL-C response locus, while emphasizing the need for larger replication studies before gene-diet associations can be inferred.

## 1 Introduction

Dyslipidemia remains a central, modifiable driver of atherosclerotic cardiovascular disease risk, and dietary change is a first-line lifestyle strategy to lower low-density lipoprotein cholesterol (LDL-C). Among the most studied dietary patterns, Mediterranean-style diets have shown cardiovascular benefit in both primary and secondary prevention settings (1, 2). However, lipid responses to dietary modification are notably heterogeneous. Under comparable guidance, the degree of improvement varies substantially, with some patients showing little benefit or even paradoxical worsening. This inter-individual variability underscores the rationale for precision nutrition approaches (3).

Nutrigenomics investigates the interaction between diet and genetic variation in shaping disease risk and therapeutic response, with the aim of explaining inter-individual variability in metabolic traits. However, much of the current evidence base remains centered on candidate-gene studies. While interactions have been reported for well-studied loci such as *APOE* and *CETP* (4, 5), evidence for other loci (e.g., *APOA5*, *ABCG5*) remains sparse, limiting inference beyond specific study settings (6, 7). Research often remains constrained to a small set of historical loci, leaving a fragmented evidence base that is difficult to consolidate. Moreover, as current evidence is heavily skewed toward specific populations, generalizability remains a critical gap (8).

To address these limitations, genome- and exome-wide approaches can support broader exploratory screening beyond restricted candidate lists. We conducted an exploratory study to examine whether genetic variation may be related to blood lipid changes in a clinical cohort receiving Mediterranean-style dietary guidance. To broaden the analysis beyond preselected candidate variants while focusing on coding-region variation captured by WES, we utilized a parallel strategy: an exploratory exome-wide nutrigenetic analysis to screen for nominal variant-level signals; and a targeted analysis of biologically plausible genes. Specifically, the ‘Plasma Lipoprotein Assembly, Remodeling, and Clearance’ pathway was selected *a priori* based on its biological relevance to lipid metabolism and dietary lipid handling. We focused primarily on LDL-C response given its central role in atherosclerotic risk, with TAG (triglycerides) analyzed as a secondary lipid trait.

## 2 Materials and methods

### 2.1 Ethics and participants

The study protocol was approved by the Ilia State University Bioethics Committee (Approval No. R/215-24, 01.07.2024) and conducted in accordance with the Declaration of Helsinki. Written informed consent was obtained from all participants, and clinical data were anonymized prior to analysis. Patients identified with elevated LDL cholesterol at an initial visit were enrolled during their subsequent clinical follow-up. Eligible individuals had completed at least 2 months of prescribed Mediterranean-style dietary guidance and had both baseline and follow-up lipid profiles available.

Inclusion criteria comprised informed consent, self-reported adherence to dietary recommendations assessed via questionnaire, availability of lipid data for baseline and follow-up time points, and euthyroid status. Exclusion criteria included lipid-lowering medication use, bariatric surgery, major weight change, diabetes mellitus, established cardiovascular disease, secondary or hereditary dyslipidemia, pregnancy or lactation, severe comorbidities (e.g., active malignancy, renal failure, or hepatic insufficiency), uncontrolled hypertension, heavy alcohol intake, or any condition impairing participation. A total of 52 participants enrolled and underwent whole-exome sequencing.

### 2.2 Clinical data

Biochemical analysis of venous blood serum was performed on a fully automated Cobas c111 clinical chemistry analyzer (Roche Diagnostics, Basel, Switzerland). Low-density lipoprotein cholesterol (LDL-C) and triglycerides (TAG) were quantified via enzymatic colorimetric methods using the Cholesterol Gen.3 kit (Ref: 7005806190) and the standard Triglycerides kit (Ref: 04657594190), respectively.

### 2.3 DNA extraction and sequencing

Genomic DNA was extracted from peripheral blood using the QIAamp DNA Blood Mini Kit (Ref: 51106, Qiagen, Hilden, Germany). DNA purity and concentration were evaluated via NanoDrop spectrophotometry (Thermo Fisher Scientific, Waltham, MA, USA); samples meeting quality control requirements were lyophilized and shipped to the sequencing facility. Whole-exome sequencing was performed by BMKGENE (Beijing, China). Libraries were prepared using the SureSelect Human All Exon V6 capture kit (Agilent Technologies, Santa Clara, CA, USA) according to manufacturer protocols and sequenced on an Illumina NovaSeq platform (Illumina, San Diego, CA, USA) generating paired-end 150-bp reads. Base calling was performed using Illumina CASAVA v1.8.

### 2.4 Lifestyle and adherence variables

Lifestyle variables were obtained using a brief self-administered follow-up questionnaire. The variables reported in the present study were limited to self-reported exercise frequency and duration, alcohol intake, cigarette smoking, and dietary adherence. Exercise volume was calculated as reported exercise sessions per week multiplied by reported average session duration. Alcohol intake and cigarette smoking were coded as binary variables. Dietary adherence was assessed using a single self-rated item asking participants to rate their overall adherence to the prescribed dietary recommendations on a 1–10 scale, with higher scores indicating greater adherence. The questionnaire was designed for clinical follow-up and was not a validated dietary intake instrument; therefore, detailed food-frequency responses were not analyzed as quantitative dietary covariates. Objective biochemical markers of dietary adherence were not collected.

### 2.5 Data processing and variant analysis

Low-quality reads were removed if they contained adaptor contamination, more than 10% undetermined bases, or more than 50% low-quality bases. Clean reads were aligned to the human reference genome GRCh38 using BWA-MEM (RRID: SCR_010910) with default parameters. Post-alignment processing followed GATK Best Practices using standard default settings, including duplicate removal via Picard (RRID: SCR_006525), base-quality score recalibration, and indel realignment. Variant calling was performed using GATK HaplotypeCaller (RRID: SCR_001876), and high-confidence variants were retained following Variant Quality Score Recalibration. Variants were annotated using ANNOVAR (RRID: SCR_012821) with default databases (9).

### 2.6 Statistical analysis

Genotype data processing and exome-wide association analyses were conducted using PLINK v2.0 (RRID: SCR_001757) (10). Downstream statistical analyses were performed in the R computing environment (version 4.1.1; RRID: SCR_001905). Data management was performed using data.table (version 1.16.4; RRID: SCR_023539) (11). Genomic annotations and gene coordinates were retrieved using biomaRt (version 2.62.1; RRID: SCR_019214) (12), and diagnostic plots were generated using qqman (version 0.1.9; RRID: SCR_024293) (13). Genomic coordinates were mapped using the Ensembl database (GRCh38/hg38 assembly, RRID: SCR_002344). All scripts used for quality control, exome-wide association analyses, targeted pathway analysis, and figure generation are publicly available on Zenodo (14).

Variants were filtered based on strict quality control criteria: minor allele frequency (MAF) > 0.01, genotype call rate > 0.90, and Hardy–Weinberg equilibrium *p* > 1 × 10^−6^. To account for population stratification, linkage disequilibrium (LD) pruning was performed (200-variant window, *r*^2^ < 0.1) and principal component analysis (PCA) was conducted. Relatedness was assessed using the KING kinship coefficient (15); one first-degree relative (kinship > 0.177) was identified and excluded to ensure sample independence prior to association testing.

Exome-wide association analyses were conducted separately for LDL-C and TAG using generalized linear models (GLM) in PLINK 2.0 under an additive genetic model. Linear regression was performed on the Winsorized change in lipid levels (mean ± 3 standard deviations) to minimize the impact of outliers while preserving interpretable effect estimates. All models were adjusted for age, sex and follow-up duration. Baseline LDL-C and TAG values were not included as covariates in the primary genetic association models because the outcomes were defined as within-person lipid change and baseline lipid levels may themselves be influenced by genetic variation. Adjusting for baseline could therefore introduce overadjustment or collider bias and attenuate genetic signals related to lipid biology. Baseline-response relationships were instead evaluated descriptively as part of the assessment of potential confounding and regression to the mean. Nominal *p*-values and Benjamini–Hochberg FDR adjusted *p*-values are reported for the prioritization of candidate loci. A *post hoc* detectable-effect-size analysis was performed to contextualize statistical power in the final analytical sample. Detectable per-allele effects were estimated for the final outcome-specific analytical samples at 80% power under an additive genetic model, using the observed variability of ΔLDL-C and ΔTAG across a range of minor allele frequencies (MAF = 0.05–0.30). Estimates were calculated for nominal *α* = 0.05 and a suggestive screening threshold of *α* = 1 × 10^−5^ and are reported in Supplementary Table S1.

### 2.7 Targeted gene analysis

In addition to the exome-wide analysis, a targeted analysis was performed on genes within the Reactome pathway “Plasma Lipoprotein Assembly, Remodeling, and Clearance” (R-HSA-174824). This pathway was selected *a priori* because it represents a curated biological process directly related to lipoprotein assembly, remodeling, receptor-mediated clearance, and triglyceride-rich lipoprotein metabolism, which were considered central to the lipid-response phenotypes analyzed in this study. Variants mapping to these genes (GRCh38 gene coordinates) were extracted from the post-QC dataset. Within each Reactome gene interval, variants were retained for Simes aggregation if at least two genotype classes were observed and the smallest genotype class included at least two individuals. For each retained variant, a linear model was fitted using the covariate structure: Δlipid ~ genotype + age + sex + follow-up duration. Follow-up duration was included because the interval between baseline and follow-up lipid assessment varied across participants. Variant-level *p*-values within each gene were then summarized using the Simes method (16). This approach tests the global null hypothesis (that at least one variant is associated) and remains valid under the linkage disequilibrium structures typical of genomic data (17). Gene-level *p*-values were subsequently adjusted for multiple comparisons using the Benjamini–Hochberg FDR.

Because pathway-based analyses may be influenced by the selected gene set, we performed a sensitivity analysis using the Blueprint Genetics Hyperlipidemia Panel (test code CA1101), a 20-gene clinical diagnostic panel for hyperlipidemia (18), as an alternative clinically anchored gene set. This panel was used to assess whether the targeted results were dependent on Reactome pathway selection, not as an independent validation set. The same variant-level modeling and Simes gene-level aggregation approach was applied, with FDR calculated across panel genes separately for each lipid outcome.

## 3 Results

### 3.1 Sequencing performance and data quality

Whole-exome sequencing produced usable data for all 52 samples, with no exclusions due to sequencing failure or insufficient quality. The mean sequencing depth across target regions was 111.9×, the proportion of bases with Q30 quality was 96.87%, and the mean mapping efficiency was approximately 99.9%. Variant calling identified approximately 135,000–156,000 SNPs per sample. Relatedness assessment identified one first-degree related pair (kinship > 0.177); one individual was excluded to ensure sample independence. Principal component analysis (PCA) on LD-pruned variants demonstrated a homogenous population structure without significant stratification outliers. After exclusion of one first-degree relative, 51 unrelated WES participants remained. Because follow-up interval and complete model covariate/outcome data were available for 48 participants, the final duration-adjusted genetic association analyses were performed in this complete-case cohort.

### 3.2 Participant characteristics

Characteristics of the 48-participant complete-case cohort used for the final duration-adjusted genetic association models are summarized in Table 1. This cohort included 27 females and 21 males. Mean age was 47.9 ± 12.9 years, and the mean follow-up interval was 4.7 ± 1.9 months. Follow-up interval was slightly longer in males than females (*p* = 0.044), supporting its inclusion as a covariate in the final genetic association models. Baseline LDL-C was 151.2 ± 26.5 mg/dL and decreased to 117.7 ± 22.2 mg/dL at follow-up, with a mean change of −33.5 ± 24.6 mg/dL. Baseline TAG was 150.5 ± 68.3 mg/dL and decreased to 131.8 ± 69.6 mg/dL at follow-up, with a mean change of −18.6 ± 58.5 mg/dL. TAG concentrations were higher in males than females at baseline and follow-up (*p* = 0.026 and *p* = 0.025, respectively), whereas lipid-response magnitudes did not differ by sex (ΔLDL-C *p* = 0.619; ΔTAG *p* = 0.903).

**Table 1:** Baseline, follow-up, lifestyle, and adherence characteristics of the complete-case genetic analysis cohort.

Variable	*N*	Overall (*n* = 48)	Female (*n* = 27)	Male (*n* = 21)	*p*-value
Age (years)	48	47.9 ± 12.9	50.4 ± 12.7	44.8 ± 12.7	0.136
Follow-up interval (months)	48	4.7 ± 1.9	4.2 ± 1.9	5.4 ± 1.9	0.044
Baseline weight (kg)	46	90.2 ± 14.7	83.6 ± 12.3	97.9 ± 13.6	<0.001
Follow-up weight (kg)	48	87.0 ± 14.4	82.6 ± 13.7	92.6 ± 13.6	0.016
Δ weight (kg)	46	−3.3 ± 6.0	−1.6 ± 3.2	−5.3 ± 7.8	0.052
Baseline LDL-C (mg/dL)	48	151.2 ± 26.5	149.4 ± 29.6	153.5 ± 22.3	0.590
Follow-up LDL-C (mg/dL)	48	117.7 ± 22.2	114.3 ± 20.7	122.0 ± 23.8	0.241
Δ LDL-C (mg/dL)	48	−33.5 ± 24.6	−35.1 ± 22.4	−31.4 ± 27.7	0.619
Baseline TAG (mg/dL)	48	150.5 ± 68.3	129.7 ± 45.2	177.1 ± 83.5	0.026
Follow-up TAG (mg/dL)	48	131.8 ± 69.6	110.1 ± 37.4	159.8 ± 90.0	0.025
Δ TAG (mg/dL)	48	−18.6 ± 58.5	−19.7 ± 29.0	−17.3 ± 83.4	0.903
Baseline TC (mg/dL)	48	223.3 ± 30.0	216.8 ± 30.3	231.7 ± 28.1	0.085
Follow-up TC (mg/dL)	48	189.3 ± 27.0	182.3 ± 22.0	198.3 ± 30.5	0.050
Δ TC (mg/dL)	48	−34.0 ± 23.2	−34.5 ± 23.5	−33.4 ± 23.4	0.873
Baseline HDL-C (mg/dL)	48	51.2 ± 11.2	55.0 ± 10.6	46.4 ± 10.2	0.007
Follow-up HDL-C (mg/dL)	48	53.7 ± 11.9	57.3 ± 11.0	49.2 ± 11.6	0.019
Δ HDL-C (mg/dL)	48	2.5 ± 6.5	2.3 ± 6.2	2.8 ± 6.9	0.777
Exercise volume (min/week)	43	247.4 ± 96.0	240.4 ± 102.3	258.2 ± 87.4	0.544
Any alcohol intake, *n* (%)	47	1/47 (2.1%)	0/27 (0.0%)	1/20 (5.0%)	0.426
Current smoking, *n* (%)	47	2/47 (4.3%)	0/27 (0.0%)	2/20 (10.0%)	0.176
Self-rated diet adherence (1–10)	46	7.6 ± 1.1	7.4 ± 1.4	7.8 ± 0.7	0.308

Continuous variables are reported as mean ± SD. Binary variables are reported as *n*/*N* (%). *N* indicates the number of participants with available data for each variable within the complete-case genetic analysis cohort. *Δ* values were calculated as follow-up minus baseline. Exercise volume was calculated as reported exercise sessions per week multiplied by reported session duration. *p*-values compare females and males and were calculated using Welch’s independent-samples *t*-test for continuous variables and Fisher’s exact test for binary variables. LDL-C, low-density lipoprotein cholesterol; TAG, triglycerides; TC, total cholesterol; HDL-C, high-density lipoprotein cholesterol.

### 3.3 Exome-wide association results

**Figure 1:**
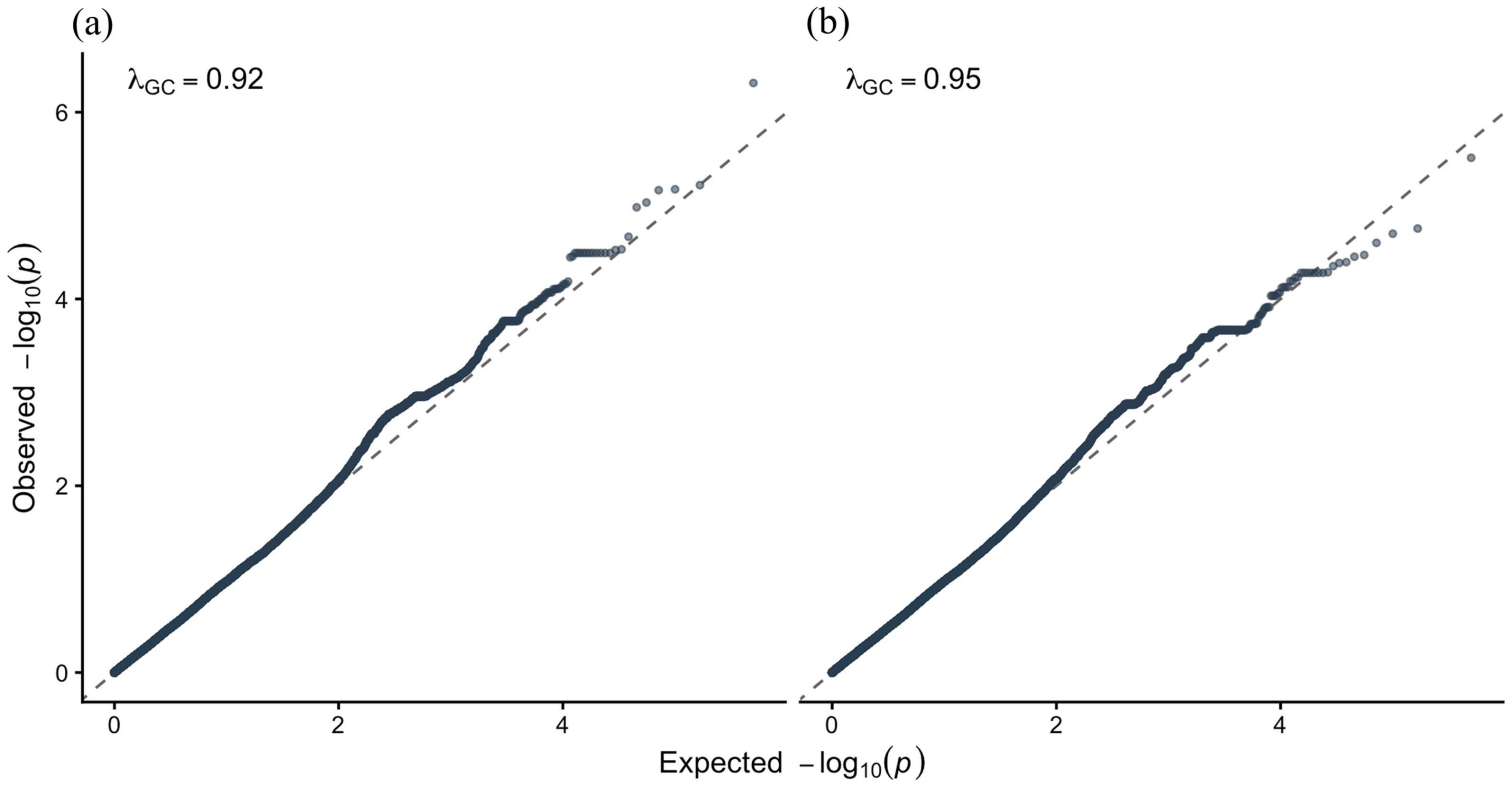
Quantile-Quantile (Q-Q) plots for the exome-wide nutrigenetic analysis of lipid response. Observed −log10(*p*) values are plotted against the expected null distribution for genetic variants modulating the response to dietary intervention. Panels represent **(a)** *Δ*LDL-C and **(b)** ΔTriglycerides (TAG), reflecting the individual variability in lipid change from baseline.

Quantile-quantile (QQ) plots of the observed versus expected *p*-values showed no evidence of genomic inflation (Figure 1), with genomic control lambda values of 0.92 for LDL-C change and 0.95 for TAG change. Additionally, principal component analysis did not indicate that population structure substantially biased the observed lipid responses (Supplementary Figure S1). Full linear model coefficients, including the effects of age, sex and follow-up duration, are provided in Supplementary Table S2.

**Figure 2:**
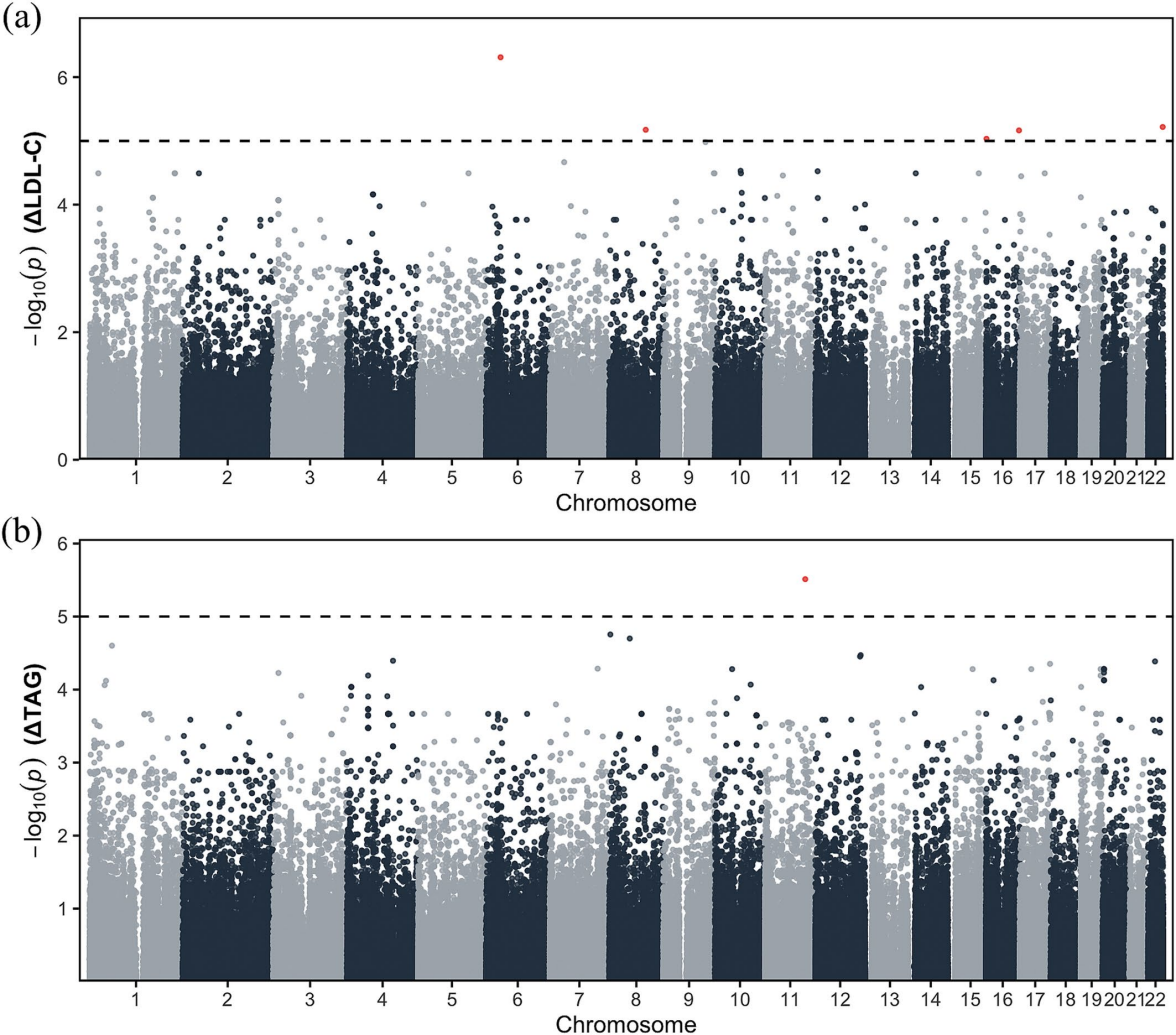
Exome-wide Manhattan plots of nutrigenetic associations with lipid response. Plots illustrate the genomic landscape of variants associated with the individual response to Mediterranean-style dietary guidance for **(a)** ΔLDL-C and **(b)** ΔTriglycerides (TAG). The dashed horizontal line indicates the suggestive significance threshold (*p* = 1 × 10^−5^).

No individual variant reached the exome-wide significance threshold (*p* < 5 × 10^−8^). This was expected given the limited sample size and detectable-effect-size estimates. At the suggestive screening threshold of *α* = 1 × 10^−5^, 80% power would require per-allele effects of approximately 30.63–64.41 mg/dL for ΔLDL-C and 49.69–104.49 mg/dL for ΔTAG across MAF values from 0.30 to 0.05 (Supplementary Table S1). Therefore, the exome-wide results are reported as exploratory nominal signals only. Nominally suggestive exome-wide signals are shown in Figure 2. For LDL-C change, the strongest nominal exome-wide signal was observed at *KIF6* (*p* = 4.87 × 10^−7^; FDR = 0.122), with additional top-ranked nominal variants reported in Supplementary Table S3. For TAG change, the strongest nominal exome-wide signal was observed at *SLC35F2* (*p* = 3.08 × 10^−6^; FDR = 0.521), with additional top-ranked nominal variants reported in Supplementary Table S4. Given the high FDR-adjusted values, these exome-wide results are reported descriptively and are not interpreted as prioritized candidate signals. Full summary statistics are available on Zenodo (14).

### 3.4 Targeted gene analysis results

In the duration-adjusted Reactome pathway analysis, the strongest gene-level signal for ΔLDL-C was observed at *APOC3* (Simes *p* = 0.000784; FDR = 0.036), while the strongest ΔTAG signal was observed at *APOB* (Simes *p* = 0.00804; FDR = 0.36) (Supplementary Table S5). The *APOB* signal did not survive FDR correction. Although *APOC3* met the pathway-level FDR threshold, variant-level inspection showed that the signal was driven primarily by rs2070669, an intronic variant with low model-specific genotype-complete sample size (*N* = 10), supporting cautious interpretation. No gene-level signal was observed for *APOE* or *LDLR* for either lipid outcome (Supplementary Table S5). Variant-level contributors to the Simes results are provided in Supplementary Table S6. In the Blueprint Genetics Hyperlipidemia Panel sensitivity analysis, *APOC3* ranked highest for ΔLDL-C and *APOB* ranked highest for ΔTAG, broadly consistent with the Reactome analysis (Supplementary Table S7).

## 4 Discussion

### 4.1 Principal findings

In this exploratory study, we examined whether genetic variation contributes to inter-individual differences in lipid response to Mediterranean-style dietary guidance. We observed nominal exome-wide signals and a targeted pathway-level *APOC3* signal for ΔLDL-C. *APOC3* met FDR correction in the targeted Reactome analysis, but variant-level inspection showed that this signal was driven primarily by a sparse intronic variant-level association. Therefore, the result is best interpreted as a hypothesis-generating *APOC3* locus-level signal rather than definitive evidence of a gene-diet association. This cautious interpretation is consistent with current nutrigenetic frameworks emphasizing exploratory prioritization and replication before clinical or mechanistic inference (19). While the absence of significant exome-wide associations is expected in exploratory settings, the absence of genomic inflation supports the technical adequacy of the association analysis, but does not eliminate the risk of false-positive findings in this small cohort.

### 4.2 Biological interpretation

The lowest nominal exome-wide *p*-values were observed at *KIF6* for ΔLDL-C and *SLC35F2* for ΔTAG. Neither signal survived multiple-testing correction, and these loci are therefore reported descriptively rather than interpreted as confirmed mechanistic candidates for dietary lipid response.

In the targeted Reactome pathway analysis, the strongest ΔLDL-C gene-level signal was observed at *APOC3*. *APOC3* inhibits lipoprotein lipase (LPL) activity and delays hepatic uptake of triglyceride-rich lipoprotein remnants, and loss-of-function variation is associated with reduced remnant cholesterol and lower LDL-C (20, 21). This makes *APOC3* biologically plausible in the context of dietary lipid response, particularly because diets rich in monounsaturated and polyunsaturated fatty acids may influence LPL activity (22). However, variant-level inspection showed that the present signal was driven primarily by rs2070669, an intronic *APOC3* variant with low model-specific genotype-complete sample size (N = 10). The intronic location does not exclude possible regulatory relevance or linkage disequilibrium with a functional variant, but the present study cannot distinguish these possibilities. Therefore, the *APOC3* result should be interpreted as a locus-level, hypothesis-generating signal requiring replication, not as evidence for a specific causal variant or confirmed gene-diet mechanism.

### 4.3 Study design and analytical strategy

To support exploratory prioritization, we employed biologically informed hypothesis restriction (23), prioritizing pathway coherence as a complement to conventional significance thresholds (24). The decision not to adjust primary genetic models for baseline lipid levels was made to avoid potential overadjustment or collider bias, because baseline lipid concentrations may partly reflect genetic variation in lipid metabolism (25, 26). However, baseline lipid values were examined descriptively in relation to lipid change to assess regression to the mean and to support cautious interpretation of the observed response patterns (Supplementary Figures S1E,F). Ultimately, combining this analytical framework with high-quality, high-depth exome sequencing (mean 111.9×) reduces the likelihood that the observed signals are driven solely by technical artifacts, although biological validation remains necessary.

The results of this study illustrate the potential pragmatic value of this biologically informed approach. When subjected to an agnostic exome-wide analysis evaluating over 187,000 variants, some nominally suggestive signals largely comprised genes lacking established biological plausibility for lipid metabolism, possibly reflecting statistical noise in the context of limited sample size and multiple testing. However, the complementary targeted pathway framework provided a middle-ground strategy. This strategy avoids the fragmented, overly restrictive nature of traditional candidate-gene studies, while reducing, but not eliminating, the statistical burden of an agnostic exome-wide scan. Because this approach uses a curated biological process, pathway-based analyses remain dependent on prior biological assumptions and may therefore introduce selection bias.

### 4.4 Strengths and limitations

A primary strength of this study is the integration of high-depth exome sequencing with a clinical dietary intervention, complemented by a biologically informed analytical framework. However, several limitations should be acknowledged.

First, the sample size was small, with 51 unrelated WES participants and 48 participants included in the final duration-adjusted complete-case genetic association models. This substantially restricts statistical power and increases the risk of both false-negative findings and false-positive nominal signals. The *post-hoc* detectable-effect-size analysis indicated that large per-allele effects would be required for adequate power in this cohort, particularly at lower minor allele frequencies and stricter alpha thresholds. Consequently, modest true effects may remain undetected, and nominal findings with elevated FDR values should be interpreted cautiously as exploratory prioritization signals rather than robust genetic associations.

Second, dietary exposure and adherence were assessed using self-reported clinical data rather than detailed quantitative intake measures or objective biomarkers. This may introduce measurement error and reduce the ability to distinguish genetic differences in dietary response from variation in exposure intensity, incomplete compliance, or other lifestyle changes. Future studies should incorporate validated dietary assessment instruments and objective markers of Mediterranean-style dietary exposure, such as plasma or erythrocyte fatty-acid profiles, omega-3 index, circulating carotenoids, urinary polyphenol metabolites, or other biomarkers matched to the dietary components under investigation. In addition, because this study did not include a randomized control group, the observed lipid changes cannot be attributed causally to Mediterranean-style dietary guidance alone. Regression to the mean, variation in adherence, follow-up duration, and concurrent lifestyle changes may also have contributed to the observed lipid responses.

Third, the use of WES defines the scope of genetic variation interrogated in this study. WES was selected to support exploratory analysis of coding and splice-proximal variation beyond a fixed candidate-SNP panel, which is particularly relevant in an underrepresented cohort with limited prior genomic characterization. However, WES does not comprehensively capture non-coding regulatory variants that are important for several lipid and nutrigenetic loci. Therefore, null findings in genes such as CETP, APOA5, APOE, or LDLR should not be interpreted as evidence against their biological relevance, but may reflect limited statistical power, incomplete capture of relevant regulatory variation, population-specific allele frequencies, or a combination of these factors.

Finally, targeted pathway analysis depends on the selected gene set. Although we used a curated Reactome pathway and performed a sensitivity analysis using a clinical hyperlipidemia gene panel, neither approach eliminates the possibility of bias from prior biological assumptions. Variant-level inspection also showed that some gene-level signals were influenced by variants with low minor allele counts or sparse genotype groups, which increases uncertainty around effect-size estimates in a small WES cohort. The pathway results should therefore be interpreted as candidate-prioritization signals rather than definitive variant-level associations. The absence of an independent replication cohort is a major limitation and precludes confirmatory biological or clinical interpretation of the nominal genetic findings.

## 5 Conclusion

This exploratory study suggests that lipid responses to dietary intervention may involve modest, distributed genetic effects that align with biologically coherent processes rather than single high-impact variants. By integrating exploratory exome-wide analyses with process-focused gene prioritization, we present a pragmatic exploratory framework for interpreting nutrigenomic intervention data under realistic constraints. These findings emphasize the potential value of biologically informed approaches for generating testable hypotheses about inter-individual dietary response.

Future efforts should focus on generating and standardizing comprehensive, consensus-driven panels of biologically plausible nutrigenetic loci, alongside the standardization of research frameworks to ensure cross-study consistency. Larger, more ancestrally diverse cohorts and precise dietary intake quantification will be essential for replicating and refining these findings. Ultimately, such work may help clarify when and how genetic information can meaningfully improve dietary response prediction.

## Data Availability

The complete analytical script and non-sensitive derived outputs used in this study are publicly available on Zenodo (DOI: https://doi.org/10.5281/zenodo.18900556). The participant-level clinical and raw genomic datasets presented in this article are not readily available because they contain sensitive human data restricted by ethical regulations and patient confidentiality agreements. However, these datasets will be made available upon reasonable request, subject to appropriate ethical approvals and data sharing agreements. Requests to access these restricted datasets should be directed to Saba Iordanishvili at saba.iordanishvili@iliauni.edu.ge.
