# Supplementary material for: Exploratory whole-exome analysis of low-density lipoprotein cholesterol and triglyceride response to Mediterranean-style dietary guidance: a focus on plasma lipoprotein pathways
Source: Front Nutr. 2026 Jul 16;13:1827989. doi: 10.3389/fnut.2026.1827989 (PMC13421421; doi:10.3389/fnut.2026.1827989)
Supplement: Supplementary file 1 [file Table_1.docx]

**Supplementary Materials**

**Title**: Exploratory Whole-Exome Analysis of Low-Density Lipoprotein Cholesterol and Triglyceride Response to Mediterranean-Style Dietary Guidance: A Focus on Plasma Lipoprotein Pathways

**Authors**: Saba Iordanishvili*, Nazibrola Chiradze, Dodo Agladze, Marine Kikvidze, Zaza Khuchua, Vincenzo Lagani and Revaz Solomonia

**Supplementary Table S1**

Post hoc detectable-effect-size analysis

| **Outcome** | N | **Alpha** | **MAF** | **Detectable β, mg/dL** |
| --- | --- | --- | --- | --- |
| ΔLDL-C | 48 | 0.05 | 0.05 | 31.13 |
| ΔLDL-C | 48 | 0.05 | 0.10 | 22.62 |
| ΔLDL-C | 48 | 0.05 | 0.20 | 16.96 |
| ΔLDL-C | 48 | 0.05 | 0.30 | 14.81 |
| ΔLDL-C | 48 | 1 × 10⁻⁵ | 0.05 | 64.41 |
| ΔLDL-C | 48 | 1 × 10⁻⁵ | 0.10 | 46.79 |
| ΔLDL-C | 48 | 1 × 10⁻⁵ | 0.20 | 35.09 |
| ΔLDL-C | 48 | 1 × 10⁻⁵ | 0.30 | 30.63 |
| ΔTAG | 47 | 0.05 | 0.05 | 50.38 |
| ΔTAG | 47 | 0.05 | 0.10 | 36.6 |
| ΔTAG | 47 | 0.05 | 0.20 | 27.45 |
| ΔTAG | 47 | 0.05 | 0.30 | 23.96 |
| ΔTAG | 47 | 1 × 10⁻⁵ | 0.05 | 104.49 |
| ΔTAG | 47 | 1 × 10⁻⁵ | 0.10 | 75.91 |
| ΔTAG | 47 | 1 × 10⁻⁵ | 0.20 | 56.93 |
| ΔTAG | 47 | 1 × 10⁻⁵ | 0.30 | 49.69 |

*Note. Detectable effect sizes were estimated at 80% power under an additive genetic model. β represents the approximate per-allele effect size in mg/dL required to detect an association for ΔLDL-C or ΔTAG at the specified minor allele frequency (MAF) and alpha threshold. Detectable effect sizes were estimated using the observed variability of each lipid-response phenotype*


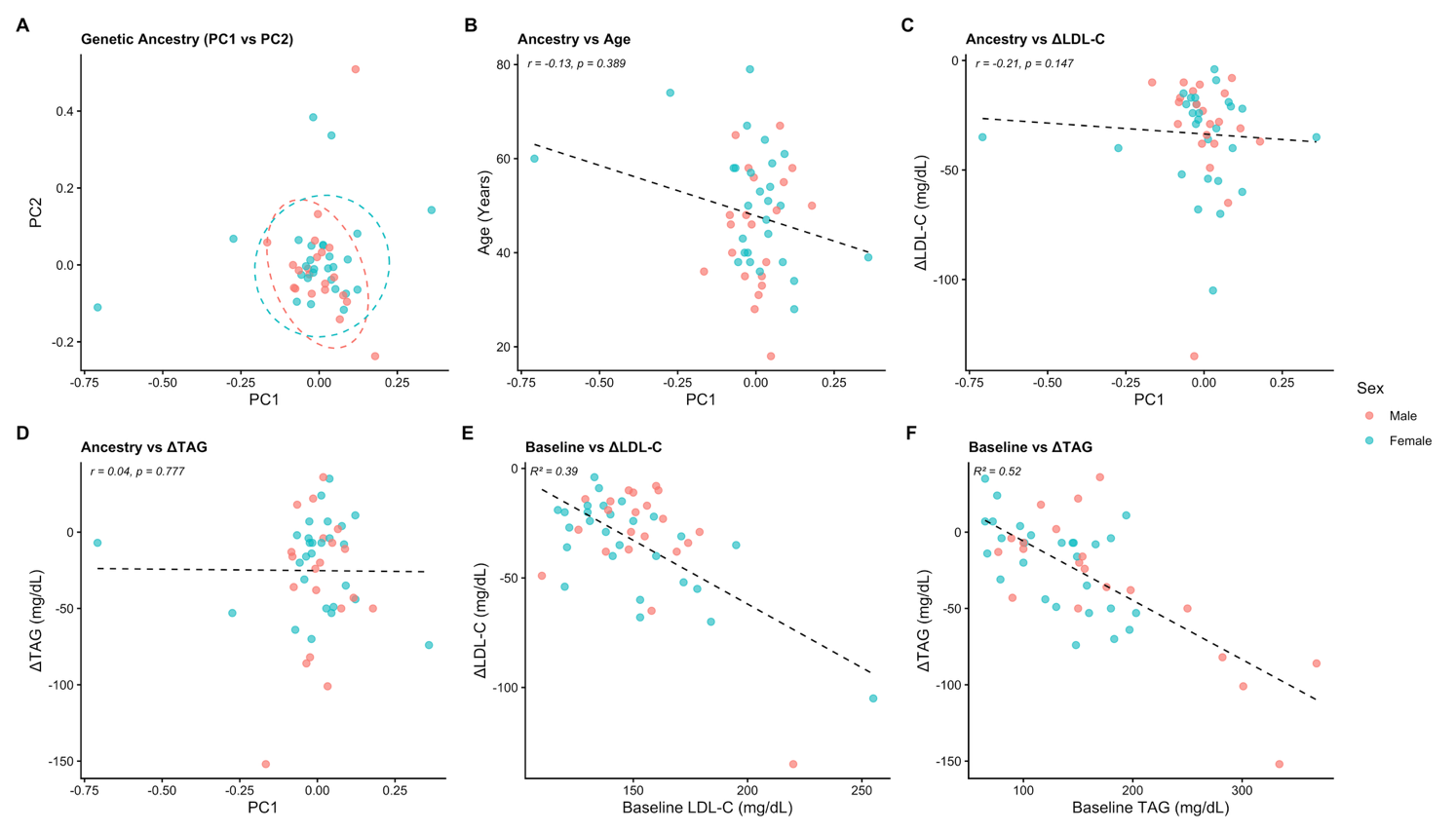


**Figure S1. Population structure and assessment of lipid response confounders.** (A) Principal component analysis (PC1 vs. PC2) color-coded by sex with 95% confidence ellipses, illustrating cohort genetic homogeneity. (B) Correlation between genetic ancestry (PC1) and participant age. (C) Relationship between PC1 and the change in LDL-C (ΔLDL-C) and (D) the change in triglycerides (ΔTAG), demonstrating that ancestry does not significantly bias the observed treatment response. (E) Association between baseline LDL-C and ΔLDL-C, and (F) baseline triglycerides and ΔTAG, showing baseline-dependence of lipid change. Dashed black lines represent linear regression trends; points are color-coded by sex (Male: pink, Female: teal). Spearman’s r, p-values, and R^2^ metrics are provided to quantify relationships.

**Supplementary Table S2**

Covariate and ancestry principal component associations with lipid response.

| Outcome | Predictor | Beta | SE | P_Value |
| --- | --- | --- | --- | --- |
| ΔLDL-C | (Intercept) | 26.105 | 14.834 | 0.0856 |
| ΔLDL-C | Age | -0.455 | 0.251 | 0.0764 |
| ΔLDL-C | Sexm | -9.147 | 6.538 | 0.169 |
| ΔLDL-C | FollowUpMonths | -6.906 | 1.655 | 0.0001 |
| ΔLDL-C | PC1 | -13.952 | 22.708 | 0.5422 |
| ΔTAG | (Intercept) | -12.956 | 26.889 | 0.6324 |
| ΔTAG | Age | -0.399 | 0.451 | 0.3814 |
| ΔTAG | Sexm | 14.92 | 11.816 | 0.2137 |
| ΔTAG | FollowUpMonths | -0.375 | 3.1 | 0.9044 |
| ΔTAG | PC1 | -7.285 | 40.931 | 0.8596 |

*Note: Linear models evaluated age, sex, follow-up duration, and PC1 in relation to ΔLDL-C and ΔTAG. PC1 was assessed as part of population-structure evaluation and was not included in the final genetic association models.*

**Supplementary Table S3**

Top variants in the Exome-wide nutrigenetic associations with LDL-C (ΔLDL-C)

| **Gene** | **Variant** | **N** | **MAF** | **MAC** | **Genotype counts** | **β** | **SE** | **P** | **FDR** |
| --- | --- | --- | --- | --- | --- | --- | --- | --- | --- |
| *KIF6* | chr6:39343872 T>C | 48 | 0.042 | 4 | 0=44; 1=4; 2=0 | -49.41 | 8.32 | 4.87 × 10^-7^ | 0.122 |
| *BRD1* | chr22:49775509 A>G | 47 | 0.298 | 28 | 0=23; 1=20; 2=4 | -18.26 | 3.52 | 6.04 × 10^-6^ | 0.403 |
| intergenic; nearest: *MATN2* (3.5 kb) | chr8:97865309 T>C | 48 | 0.177 | 17 | 0=33; 1=13; 2=2 | -20.42 | 3.97 | 6.69 × 10^-6^ | 0.403 |
| *ZFPM1* | chr16:88485962 A>G | 48 | 0.448 | 43 | 0=13; 1=27; 2=8 | -17.51 | 3.41 | 6.84 × 10^-6^ | 0.403 |
| *TELO2* | chr16:1499845 C>T | 46 | 0.022 | 2 | 0=44; 1=2; 2=0 | -62.65 | 12.34 | 9.27 × 10^-6^ | 0.403 |
| *SNX30* | chr9:112804670 T>A | 48 | 0.073 | 7 | 0=41; 1=7; 2=0 | -31.10 | 6.21 | 1.04 × 10^-5^ | 0.403 |
| *VPS41* | chr7:38817812 C>G | 48 | 0.031 | 3 | 0=45; 1=3; 2=0 | -50.93 | 10.65 | 2.16 × 10^-5^ | 0.403 |
| *DNA2* | chr10:68471962 C>G | 46 | 0.022 | 2 | 0=44; 1=2; 2=0 | -60.42 | 12.82 | 2.94 × 10^-5^ | 0.403 |
| *ANO2* | chr12:5945111 G>A | 48 | 0.208 | 20 | 0=30; 1=16; 2=2 | -19.12 | 4.08 | 2.99 × 10^-5^ | 0.403 |
| *MDS2* | chr1:23627676 C>A | 48 | 0.021 | 2 | 0=46; 1=2; 2=0 | -60.24 | 12.94 | 3.22 × 10^-5^ | 0.403 |

*Note. Variants are ranked by nominal p value. ΔLDL-C was defined as follow-up minus baseline LDL-C (mg/dL). Association testing used an additive genetic model (PLINK 2.0 GLM) adjusted for age, sex, and follow-up duration. β indicates change in ΔLDL-C per additional copy of the counted allele (mg/dL). MAF, MAC, and genotype counts were calculated in the analyzed sample for each variant. FDR values are Benjamini–Hochberg adjusted across all tested variants.*

**Supplementary Table S4**

Top variants in the Exome-wide nutrigenetic associations with triglycerides (ΔTAG)

| **Gene** | **Variant** | **N** | **MAF** | **MAC** | **Genotype counts** | **β** | **SE** | **P** | **FDR** |
| --- | --- | --- | --- | --- | --- | --- | --- | --- | --- |
| *SLC35F2* | chr11:107908678 G>A | 47 | 0.043 | 4 | 0=43; 1=4; 2=0 | -84.23 | 15.66 | 3.08 × 10^-6^ | 0.521 |
| *CSMD1* | chr8:3158034 A>G | 47 | 0.043 | 4 | 0=43; 1=4; 2=0 | -78.43 | 16.19 | 1.76 × 10^-5^ | 0.521 |
| *XKR4* | chr8:55142136 C>T | 47 | 0.191 | 18 | 0=30; 1=16; 2=1 | 39.98 | 8.32 | 2 × 10^-5^ | 0.521 |
| *C1orf87* | chr1:60041090 G>A | 45 | 0.044 | 4 | 0=41; 1=4; 2=0 | -76.90 | 16.14 | 2.51 × 10^-5^ | 0.521 |
| *P2RX7* | chr12:121155552 C>A | 43 | 0.047 | 4 | 0=39; 1=4; 2=0 | -63.41 | 13.49 | 3.38 × 10^-5^ | 0.521 |
| *CCDC60* | chr12:119489054 G>A | 47 | 0.032 | 3 | 0=44; 1=3; 2=0 | -84.87 | 18.34 | 3.53 × 10^-5^ | 0.521 |
| *NUDT6* | chr4:122922561 T>C | 47 | 0.149 | 14 | 0=34; 1=12; 2=1 | -40.11 | 8.75 | 4.03 × 10^-5^ | 0.521 |
| *AP1B1* | chr22:29339658 C>T | 46 | 0.054 | 5 | 0=43; 1=1; 2=2 | -48.90 | 10.65 | 4.12 × 10^-5^ | 0.521 |
| *SLC38A10* | chr17:81251292 C>G | 47 | 0.064 | 6 | 0=41; 1=6; 2=0 | -62.79 | 13.78 | 4.45 × 10^-5^ | 0.521 |
| *IMPDH1* | chr7:128400698 C>T | 45 | 0.322 | 29 | 0=23; 1=15; 2=7 | -25.38 | 5.60 | 5.18 × 10^-5^ | 0.521 |

*Note. Variants are ranked by nominal p value. ΔTAG was defined as follow-up minus baseline triglycerides (mg/dL). Association testing used an additive genetic model (PLINK 2.0 GLM) adjusted for age, sex, and follow-up duration. β indicates change in ΔTAG per additional copy of the counted allele (mg/dL). MAF, MAC, and genotype counts were calculated in the analyzed sample for each variant. FDR values are Benjamini–Hochberg adjusted across all tested variants.*

**Supplementary Table S5**

Targeted gene-level nutrigenetic associations for lipid response

| LDL-C | | | | TAG | | | |
| --- | --- | --- | --- | --- | --- | --- | --- |
| Gene | N_Vars | Simes_P | FDR | Gene | N_Vars | Simes_P | FDR |
| *APOC3* | 5 | 0.000784 | 0.0361 | *APOB* | 31 | 0.008041 | 0.3573 |
| *AP2A2* | 27 | 0.024057 | 0.3958 | *ABCG1* | 16 | 0.020805 | 0.3573 |
| *APOC2* | 3 | 0.033017 | 0.3958 | *MYLIP* | 5 | 0.023299 | 0.3573 |
| *AP2A1* | 16 | 0.041779 | 0.3958 | *AP2S1* | 12 | 0.111465 | 0.9350 |
| *A2M* | 24 | 0.043022 | 0.3958 | *AP2M1* | 23 | 0.121711 | 0.9350 |
| *AP2B1* | 9 | 0.067792 | 0.4697 | *APOBR* | 6 | 0.121961 | 0.9350 |
| *AMN* | 6 | 0.071480 | 0.4697 | *SOAT2* | 9 | 0.161483 | 0.9484 |
| *ALB* | 7 | 0.204277 | 0.9789 | *APOA1* | 9 | 0.183786 | 0.9484 |
| *RPS27A* | 2 | 0.237369 | 0.9789 | *LDLRAP1* | 2 | 0.185555 | 0.9484 |
| *SAR1B* | 2 | 0.239926 | 0.9789 | *APOC3* | 32 | 0.237587 | 0.9654 |
| *AP2S1* | 3 | 0.263061 | 0.9789 | *APOA5* | 5 | 0.264536 | 0.9654 |
| *APOF* | 4 | 0.268104 | 0.9789 | *APOF* | 4 | 0.265028 | 0.9654 |
| *PRKACA* | 12 | 0.320576 | 0.9789 | *MTTP* | 39 | 0.272826 | 0.9654 |
| *SOAT1* | 12 | 0.338220 | 0.9789 | *LPA* | 3 | 0.323840 | 0.9861 |
| *PRKACB* | 2 | 0.357694 | 0.9789 | *PRKACG* | 37 | 0.350963 | 0.9861 |
| *APOE* | 23 | 0.367130 | 0.9789 | *APOA4* | 5 | 0.388020 | 0.9861 |
| *LIPA* | 9 | 0.420963 | 0.9789 | *LDLR* | 2 | 0.430942 | 0.9861 |
| *AP2M1* | 9 | 0.433008 | 0.9789 | *PRKACA* | 8 | 0.431446 | 0.9861 |
| *LIPG* | 37 | 0.449967 | 0.9789 | *ANGPTL4* | 32 | 0.498592 | 0.9861 |
| *LPA* | 4 | 0.513762 | 0.9789 | *SOAT1* | 10 | 0.530658 | 0.9861 |
| *PRKACG* | 6 | 0.582590 | 0.9789 | *LMF1* | 9 | 0.537787 | 0.9861 |
| *ANGPTL4* | 24 | 0.586859 | 0.9789 | *RPS27A* | 6 | 0.589165 | 0.9861 |
| *APOB* | 2 | 0.590023 | 0.9789 | *NCEH1* | 12 | 0.601812 | 0.9861 |
| *MBTPS1* | 3 | 0.603282 | 0.9789 | *AP2A1* | 35 | 0.606635 | 0.9861 |
| *APOC1* | 32 | 0.620794 | 0.9789 | *APOC2* | 7 | 0.608232 | 0.9861 |
| *SOAT2* | 1 | 0.641069 | 0.9789 | *LMF2* | 6 | 0.637856 | 0.9861 |
| *APOBR* | 6 | 0.680373 | 0.9789 | *APOC1* | 42 | 0.664235 | 0.9861 |
| *ABCG1* | 19 | 0.682643 | 0.9789 | *A2M* | 24 | 0.688757 | 0.9861 |
| *NCEH1* | 4 | 0.683763 | 0.9789 | *MBTPS1* | 28 | 0.707371 | 0.9861 |
| *ANGPTL3* | 39 | 0.751460 | 0.9789 | *APOE* | 4 | 0.710964 | 0.9861 |
| *LSR* | 23 | 0.778431 | 0.9789 | *ANGPTL3* | 2 | 0.727907 | 0.9861 |
| *APOC4* | 7 | 0.798142 | 0.9789 | *APOC4* | 3 | 0.746108 | 0.9861 |
| *APOA1* | 39 | 0.804636 | 0.9789 | *LSR* | 7 | 0.776606 | 0.9861 |
| *LPL* | 8 | 0.809894 | 0.9789 | *LIPC* | 11 | 0.801578 | 0.9861 |
| *LIPC* | 5 | 0.813182 | 0.9789 | *AMN* | 31 | 0.808704 | 0.9861 |
| *MYLIP* | 9 | 0.831676 | 0.9789 | *LIPA* | 1 | 0.830926 | 0.9861 |
| *LMF1* | 31 | 0.846833 | 0.9789 | *ABCA1* | 23 | 0.882691 | 0.9861 |
| *MTTP* | 11 | 0.891649 | 0.9789 | *PRKACB* | 4 | 0.889877 | 0.9861 |
| *LDLRAP1* | 59 | 0.908038 | 0.9789 | *LPL* | 3 | 0.902464 | 0.9861 |
| *ANGPTL8* | 10 | 0.919614 | 0.9789 | *ALB* | 9 | 0.925493 | 0.9861 |
| *ABCA1* | 6 | 0.923961 | 0.9789 | *LCAT* | 19 | 0.942414 | 0.9861 |
| *LCAT* | 4 | 0.924986 | 0.9789 | *LIPG* | 22 | 0.951991 | 0.9861 |
| *LMF2* | 35 | 0.948918 | 0.9789 | *AP2A2* | 59 | 0.953234 | 0.9861 |
| *APOA4* | 43 | 0.956134 | 0.9789 | *AP2B1* | 3 | 0.978910 | 0.9861 |
| *APOA5* | 31 | 0.967159 | 0.9789 | *SAR1B* | 7 | 0.985635 | 0.9861 |
| *LDLR* | 7 | 0.978876 | 0.9789 | *ANGPTL8* | 1 | 0.986125 | 0.9861 |

*Note. Genes are ranked by their unadjusted Simes p-value for both low-density lipoprotein cholesterol (LDL-C) and triglycerides (TAG). The targeted analysis evaluated genes within the Reactome pathway "Plasma Lipoprotein Assembly, Remodeling, and Clearance". Variant-level models were adjusted for age, sex, and follow-up duration. N_Vars indicates the number of filtered variants analyzed within each gene locus. Simes_P represents the unadjusted gene-level p-value summarized using the Simes method. FDR indicates the Benjamini–Hochberg false discovery rate-adjusted p-value calculated separately for each lipid outcome.*

**Supplementary Table S6. Variant-level association results underlying pathway-level Simes signals.**

| Outcome | Gene | Variant | rsID | Variant annotation | MAF | MAC | Genotype.counts | N | β (SE) | P |
| --- | --- | --- | --- | --- | --- | --- | --- | --- | --- | --- |
| ΔLDL-C | *APOC3* | 11:116830406 C>G | rs2070669 | intronic | 0.400 | 8 | 0=4; 1=0; 2=6 | 10 | 22.83 (2.24) | 1.57 x 10^-4^ |
| ΔLDL-C | *APOC3* | 11:116832955 G>T | rs4225 | UTR3 | 0.396 | 38 | 0=18; 1=22; 2=8 | 48 | -5.48 (3.80) | 0.157 |
| ΔLDL-C | *APOC3* | 11:116832924 G>C | rs5128 | UTR3 | 0.125 | 12 | 0=39; 1=6; 2=3 | 48 | -2.03 (5.00) | 0.687 |
| ΔLDL-C | *APOC3* | 11:116830437 T>G | rs2070668 | intronic | 0.220 | 11 | 0=4; 1=3; 2=18 | 25 | -1.40 (5.91) | 0.816 |
| ΔLDL-C | *APOC3* | 11:116830819 T>C | rs4520 | p.G34G | 0.271 | 26 | 0=27; 1=16; 2=5 | 48 | 0.58 (4.14) | 0.888 |
| ΔTAG | *APOB* | 2:21015452 C>T | rs142448733 | p.S1142S | 0.021 | 2 | 0=0; 1=2; 2=45 | 47 | 92.39 (23.16) | 2.59 x 10^-4^ |
| ΔTAG | *APOB* | 2:21042269 C>A | rs661665 | intronic | 0.478 | 44 | 0=14; 1=20; 2=12 | 46 | 15.35 (7.03) | 0.035 |
| ΔTAG | *APOB* | 2:21018633 A>G | rs12713956 | intronic | 0.143 | 4 | 0=2; 1=0; 2=12 | 14 | -26.98 (18.15) | 0.171 |
| ΔTAG | *APOB* | 2:21042646 A>T | rs12714264 | intronic | 0.213 | 20 | 0=2; 1=16; 2=29 | 47 | -12.82 (9.38) | 0.179 |
| ΔTAG | *APOB* | 2:21014914 T>G | rs2854725 | intronic | 0.120 | 11 | 0=0; 1=11; 2=35 | 46 | -16.47 (12.12) | 0.182 |
| ΔTAG | *APOB* | 2:21011100 T>C | rs533617 | p.H1923R | 0.043 | 4 | 0=0; 1=4; 2=43 | 47 | 23.10 (18.86) | 0.227 |
| ΔTAG | *APOB* | 2:21028168 T>G | rs570877 | intronic | 0.117 | 11 | 0=36; 1=11; 2=0 | 47 | 15.18 (12.55) | 0.233 |
| ΔTAG | *APOB* | 2:21037729 G>A | rs550619 | intronic | 0.113 | 9 | 0=33; 1=5; 2=2 | 40 | 14.27 (11.83) | 0.236 |
| ΔTAG | *APOB* | 2:21018872 C>T | rs12720828 | intronic | 0.266 | 25 | 0=5; 1=15; 2=27 | 47 | -9.02 (7.80) | 0.254 |
| ΔTAG | *APOB* | 2:21023434 G>C | rs12714102 | intronic | 0.128 | 12 | 0=0; 1=12; 2=35 | 47 | -13.97 (12.34) | 0.264 |
| ΔTAG | *APOB* | 2:21008652 G>A | rs676210 | p.P2739L | 0.255 | 24 | 0=2; 1=20; 2=25 | 47 | 10.36 (9.34) | 0.274 |
| ΔTAG | *APOB* | 2:21014672 G>A | rs673548 | intronic | 0.255 | 24 | 0=2; 1=20; 2=25 | 47 | 10.36 (9.34) | 0.274 |
| ΔTAG | *APOB* | 2:21002409 C>T | rs1042034 | p.S4338N | 0.255 | 24 | 0=25; 1=20; 2=2 | 47 | -10.36 (9.34) | 0.274 |
| ΔTAG | *APOB* | 2:21001973 C>T | rs138421941 | p.A4483A | 0.021 | 2 | 0=0; 1=2; 2=45 | 47 | 27.93 (26.48) | 0.298 |
| ΔTAG | *APOB* | 2:21041028 G>A | rs1367117 | p.T98I | 0.223 | 21 | 0=6; 1=9; 2=32 | 47 | -7.80 (7.75) | 0.320 |
| ΔTAG | *APOB* | 2:21028042 G>A | rs679899 | p.A618V | 0.468 | 44 | 0=12; 1=26; 2=9 | 47 | 7.68 (8.00) | 0.343 |
| ΔTAG | *APOB* | 2:21014366 A>T | rs3749054 | intronic | 0.255 | 24 | 0=5; 1=14; 2=28 | 47 | -7.24 (7.85) | 0.362 |
| ΔTAG | *APOB* | 2:21002881 C>T | rs1042031 | p.E4181K | 0.266 | 25 | 0=5; 1=15; 2=27 | 47 | -5.27 (7.89) | 0.507 |
| ΔTAG | *APOB* | 2:21004511 C>G | rs1800479 | intronic | 0.266 | 25 | 0=5; 1=15; 2=27 | 47 | -5.27 (7.89) | 0.507 |
| ΔTAG | *APOB* | 2:21009323 G>A | rs693 | p.T2515T | 0.340 | 32 | 0=7; 1=18; 2=22 | 47 | 4.84 (7.46) | 0.520 |
| ΔTAG | *APOB* | 2:21026844 C>T | rs12691202 | p.V730I | 0.021 | 2 | 0=0; 1=2; 2=45 | 47 | -12.09 (27.88) | 0.667 |
| ΔTAG | *APOB* | 2:21040767 T>G | rs531819 | intronic | 0.379 | 22 | 0=15; 1=6; 2=8 | 29 | 2.68 (7.64) | 0.729 |
| ΔTAG | *APOB* | 2:21032501 T>C |  | p.H402R | 0.021 | 2 | 0=0; 1=2; 2=45 | 47 | -9.02 (27.83) | 0.748 |
| ΔTAG | *APOB* | 2:21003380 A>G | rs12691188 | intronic | 0.022 | 2 | 0=0; 1=2; 2=44 | 46 | -6.41 (28.33) | 0.822 |
| ΔTAG | *APOB* | 2:21002613 C>G | rs1801702 | p.R4270T | 0.021 | 2 | 0=0; 1=2; 2=45 | 47 | -4.96 (28.37) | 0.862 |
| ΔTAG | *APOB* | 2:21008720 G>A | rs6413458 | p.I2716I | 0.021 | 2 | 0=0; 1=2; 2=45 | 47 | -4.96 (28.37) | 0.862 |
| ΔTAG | *APOB* | 2:21020122 C>T |  | intronic | 0.021 | 2 | 0=0; 1=2; 2=45 | 47 | -2.08 (26.54) | 0.938 |
| ΔTAG | *APOB* | 2:21022941 G>A | rs1801700 | p.N902N | 0.032 | 3 | 0=0; 1=3; 2=44 | 47 | 1.67 (22.80) | 0.942 |
| ΔTAG | *APOB* | 2:21001981 C>T | rs1801695 | p.A4481T | 0.085 | 8 | 0=0; 1=8; 2=39 | 47 | 0.65 (15.09) | 0.966 |
| ΔTAG | *APOB* | 2:21022495 A>G | rs3791981 | intronic | 0.053 | 4 | 0=0; 1=4; 2=34 | 38 | -0.24 (20.16) | 0.990 |
| ΔTAG | *APOB* | 2:21016789 T>C | rs12720820 | intronic | 0.400 | 8 | 0=3; 1=2; 2=5 | 10 | 0.06 (9.07) | 0.995 |

*Note. The table shows the variants included in the Simes gene-level calculation for the prioritized pathway-level signals: APOC3 for ΔLDL-C and APOB for ΔTAG. Variant-level association models were adjusted for age, sex, and follow-up duration. N indicates the variant-specific model sample size after excluding participants with missing lipid outcome, covariate, or genotype data for that variant. Genotype counts are reported for the same model-included participants and therefore sum to N. MAF and MAC were calculated within the variant-specific model sample. Variant annotation provides a compact annotation of variant class or protein-level annotation, with non-coding variants labeled by region and exonic synonymous variants shown using p. notation.*

**Supplementary Table S7.**

Clinical hyperlipidemia gene-panel sensitivity analysis

| LDL-C | | | | TAG | | | |
| --- | --- | --- | --- | --- | --- | --- | --- |
| Gene | N_Vars | Simes_P | FDR | Gene | N_Vars | Simes_P | FDR |
| *APOC3* | 5 | 0.001 | 0.016 | *APOB* | 31 | 0.003 | 0.063 |
| *APOE* | 4 | 0.140 | 0.951 | *ALMS1* | 31 | 0.031 | 0.311 |
| *LPL* | 23 | 0.332 | 0.951 | *LMF1* | 32 | 0.173 | 0.751 |
| *LDLR* | 24 | 0.377 | 0.951 | *GPIHBP1* | 2 | 0.222 | 0.751 |
| *ABCG8* | 37 | 0.381 | 0.951 | *APOC3* | 5 | 0.260 | 0.751 |
| *APOC2* | 8 | 0.400 | 0.951 | *APOA1* | 4 | 0.265 | 0.751 |
| *APOB* | 31 | 0.431 | 0.951 | *PCSK9* | 23 | 0.315 | 0.751 |
| *ALMS1* | 31 | 0.463 | 0.951 | *APOA5* | 5 | 0.333 | 0.751 |
| *ABCG5* | 21 | 0.542 | 0.951 | *ABCG8* | 37 | 0.338 | 0.751 |
| *CYP27A1* | 4 | 0.656 | 0.951 | *GPD1* | 9 | 0.442 | 0.858 |
| *GPD1* | 9 | 0.679 | 0.951 | *LDLRAP1* | 8 | 0.472 | 0.858 |
| *GPIHBP1* | 2 | 0.740 | 0.951 | *LIPA* | 42 | 0.700 | 0.968 |
| *APOA5* | 6 | 0.765 | 0.951 | *ABCG5* | 21 | 0.716 | 0.968 |
| *PCSK9* | 24 | 0.786 | 0.951 | *APOC2* | 7 | 0.735 | 0.968 |
| *APOA1* | 4 | 0.815 | 0.951 | *LDLR* | 24 | 0.785 | 0.968 |
| *LDLRAP1* | 8 | 0.823 | 0.951 | *CYP27A1* | 4 | 0.787 | 0.968 |
| *CREB3L3* | 8 | 0.894 | 0.951 | *CREB3L3* | 8 | 0.906 | 0.968 |
| *LIPA* | 43 | 0.930 | 0.951 | *APOE* | 3 | 0.955 | 0.968 |
| *ABCA1* | 59 | 0.944 | 0.951 | *ABCA1* | 59 | 0.966 | 0.968 |
| *LMF1* | 32 | 0.951 | 0.951 | *LPL* | 22 | 0.968 | 0.968 |

*Note. This sensitivity analysis evaluated genes included in an existing clinical hyperlipidemia gene panel. Variant-level models were adjusted for age, sex, and follow-up duration before gene-level p-values were summarized using the Simes method. N_Vars indicates the number of filtered variants analyzed within each gene locus. Simes_P represents the unadjusted gene-level p-value. FDR indicates the Benjamini–Hochberg false discovery rate-adjusted p-value calculated across panel genes separately for each lipid outcome*
